# Qualität in der Befundung von Kopf- und Halssonographien an Universitätskliniken – eine Stichprobe

**DOI:** 10.1007/s00106-020-00989-9

**Published:** 2021-01-13

**Authors:** J. Künzel, A. Bozzato, B. P. Ernst, T. Fuhrmann, I. Ugele, C. Scherl, M. Schapher, G. F. Volk, N. Mansour, A. Knopf, C. Bohr, K.-F. Hamann

**Affiliations:** 1 Klinik und Poliklinik für Hals‑Nasen‑Ohren-Heilkunde, Universitätsklinikum Regensburg, Franz-Josef-Strauß-Allee 11, 93053 Regensburg, Deutschland; 2 Klinik und Poliklinik für Hals‑, Nasen‑, Ohrenheilkunde, Universitätsklinikum des Saarlandes, Homburg, Deutschland; 3 Hals‑, Nasen‑, Ohrenklinik und Poliklinik, Universitätsmedizin Mainz, Mainz, Deutschland; 4 Fachbereich Ingenieur- und Naturwissenschaften, Hochschule Merseburg, Merseburg, Deutschland; 5 Klinik für Hals-Nasen-Ohren-Heilkunde, Kopf- und Halschirurgie, Universitätsmedizin Mannheim, Mannheim, Deutschland; 6 Hals-Nasen-Ohren-Klinik, Kopf- und Halschirurgie, Universitätsklinikum Erlangen, Erlangen, Deutschland; 7 Klinik für Hals‑, Nasen- und Ohrenheilkunde, Universitätsklinikum Jena, Jena, Deutschland; 8 Klinik für Hals‑, Nasen- und Ohrenheilkunde, Universitätsklinikum Freiburg, Freiburg, Deutschland; 9 HNO-Klinik Dr. Gaertner, München, Deutschland

**Keywords:** Digitale Befunddokumentation, Ultraschallausbildung, Strukturierte Befundung, Befundqualität, Befundvollständigkeit, Digital documentation of findings, Ultrasound training, Structured reporting, Quality of findings, Completeness of findings

## Abstract

**Hintergrund:**

Die Ultraschalldiagnostik gilt für den Radiologen, Hals-Nasen-Ohren-Arzt (HNO) oder Mund-Kiefer-Gesichts-Chirurgen als Standard in der Abklärung zahlreicher Pathologien. Es besteht ein Konsens, dass die digitale Dokumentation heute dringend notwendig ist, um die Qualität der sonographischen Dokumentationen zu verbessern und zu standardisieren. Es häufen sich Publikationen zur Implementierung standardisierter Befunddokumentation einschließlich der Kopf- und Halssonographie.

**Ziel der Arbeit:**

Die vorliegende Arbeit zielt darauf ab, die Qualität von routinemäßig angefertigten Kopf- und Halssonographiebefunden nach Kriterien der Kassenärztlichen Vereinigung (KV) Bayern an einer Auswahl deutscher HNO-Universitätskliniken stichprobenartig zu ermitteln.

**Material und Methoden:**

Insgesamt wurden retrospektiv 70 zufällig ausgewählte, anonymisierte schriftliche Befunde einschließlich Bildmaterial von insgesamt 7 HNO-Universitätskliniken stichprobenartig nach KV-Kriterien durch einen erfahrenen Prüfer der KV Bayern ausgewertet und deskriptiv analysiert.

**Ergebnisse:**

Von 70 Befunden konnten 69 ausgewertet werden. Die Dokumentationsvollständigkeit lag im Mittel bei 80,6 %. Neun Befunde waren vollständig korrekt dokumentiert (13 %). Die Dokumentationsvollständigkeit der einzelnen Kliniken lag zwischen 68,1 % und 93 %. Mit 88,5 % vs. 75 % erbrachte eine strukturierte Befundung eine höhere Befundvollständigkeit. In 75 % der Fälle verfügten die Kliniken mit strukturiertem Befund auch über digitale Dokumentationslösungen.

**Schlussfolgerung:**

Die Vollständigkeit und Qualität von routinemäßig angefertigten Kopf- und Halssonographiebefunden an einer Auswahl von HNO-Universitätskliniken ist insgesamt optimierbar. Die Implementierung strukturierter Befundmasken und die Umstellung der analogen Dokumentation auf digitale Lösungen sowie Vernetzung mit dem Klinikinformationssystem (KIS) und Bildarchivierungs- und Kommunikationssystem (PACS) sollte weiter vorangetrieben werden. Darüber hinaus sind leitende Ärzte dazu angehalten, die Befundqualität unerfahrener Kollegen regelmäßig zu prüfen und im Rahmen der Facharztausbildung auf die Erfüllung entsprechender Standards wie der KV-Ultraschallvereinbarung hinzuarbeiten.

## Abstract (en)

**Background:**

Ultrasound diagnostics are widely used and are standard for radiologists, otolaryngologists, and oral and maxillofacial surgeons in the diagnostic work-up of various pathologies. There is agreement that digital documentation is urgently needed at present to improve and standardize the quality of sonographic documentation. There are more and more publications on the implementation of standardized documentation of findings in imaging diagnostics, including head and neck sonography.

**Objective:**

The present work aims to determine the quality of routine head and neck sonography findings on a random basis, according to the criteria of the Bavarian Association of Statutory Health Insurance Physicians (KVB) at a selection of German university otolaryngology departments (ENT).

**Materials and methods:**

A total of 70 randomly selected anonymized written findings including image documentation from seven ENT departments were retrospectively analyzed by an experienced KVB examiner concerning fulfilment of KVB criteria. The data were evaluated descriptively.

**Results:**

Of the 70 reports, 69 were eligible for evaluation. The average documentation completeness was 80.6%. A total of 9 findings were correctly documented in full (13%). The documentation completeness of the individual departments was sorted in ascending order from 68.1% to 93%. With 88.5% vs. 75%, the hospitals with a structured report showed a higher level of completeness. In 75% of the cases the hospitals with structured reports also had digital solutions for reporting and image archiving.

**Conclusion:**

In general, there is potential for optimization regarding the completeness and quality of routinely prepared head and neck sonography findings at the selected university ENT departments. The implementation of structured reporting masks and the conversion of analogue documentation into digital solutions as well as digital networking with the hospital information systems, picture archiving and communication systems should be promoted. Supervision by senior doctors is required to ensure the quality of findings of inexperienced colleagues and to help to achieve standards in reporting.

## Hintergrund und Fragestellung

Die Ultraschalldiagnostik genießt eine weite Verbreitung und gilt für den Radiologen, Hals-Nasen-Ohren-Arzt oder Mund-Kiefer-Gesichts-Chirurgen als Standard in der primären Abklärung diverser Pathologien. Insbesondere in der Tumornachsorge hat die Ultraschalldiagnostik ihren besonderen Stellenwert. Mitte der 1980er-Jahre wurde die Sektion „Kopf-Hals“ der Deutschen Gesellschaft für Ultraschall in der Medizin (DEGUM) gegründet. Mitte der 1990er-Jahre wurde die Ultraschalldiagnostik im Kopf-Hals-Bereich dann fester Bestandteil der Assistentenausbildung. In der HNO-Facharzt-Weiterbildung werden über 200 Ultraschalluntersuchungen des Kopf-Hals-Bereichs mit entsprechender Dokumentation gefordert. Zusätzlich werden aktuelle Ausbildungsstandards von der DEGUM vorgeschlagen (https://www.degum.de), welche neben separat belegbaren Kursen auf Basis des DEGUM-Ausbildungscurriculums die Zertifizierung über einzelne Kompetenzstufen anbietet. Im Jahr 2018 publizierten Heyduck et al. die Ergebnisse einer deutschlandweiten Umfrage zur Wertigkeit der Ultraschalldiagnostik im Kopf- und Halsbereich [9]. Vor allem die Weichteil- und onkologische Diagnostik hatten sowohl bei Kollegen in der Niederlassung als auch in den Kliniken einen hohen Stellenwert. Die Auswertung ergab, dass niedergelassene Kollegen im Vergleich zu den Kliniken bereits 2018 einen höheren Grad der Digitalisierung erreichten. Es erfolgte bereits zu diesem Zeitpunkt zu 55 % eine komplett digitale Dokumentation, hingegen bei 70 % der Befragten in der Klinik weiterhin eine manuelle Dokumentation mit Ausdruck der Befunde. Die digitale Dokumentation erscheint wichtig, um die Qualität der sonographischen Dokumentationen zu verbessern und zu standardisieren, denn nur so sind Befunde in angemessener Qualität archivierbar, abrufbar und können beispielsweise im Rahmen von Tumorkonferenzen sinnvoll zur Entscheidungsfindung hinzugezogen werden. Die moderne Informationstechnologie (KIS, PACS) und die elektronische Patientenakte setzen die digitale Integration von Bild- und Befunddaten in medizinische Informationssysteme voraus. Die Einbindung von Befunden anderer bildgebender Verfahren wie Computertomographie (CT) und Magnetresonanztomographie (MRT) sind hier als Maßstab zu betrachten [9]. Zuletzt häuften sich Publikationen, welche die Implementierung standardisierter Befunddokumentation ebenfalls für die Kopf- und Halssonographie fordern [1, 3, 5, 10, 17–19]. Ernst et al. zeigten, dass die strukturierte digitale Befunderhebung im Vergleich zur handschriftlichen in puncto Vollständigkeit, Lesbarkeit und Zeiteffizienz nach einer entsprechenden Implementierungsphase überlegen ist [5]. Die vorliegende Arbeit ermittelt stichprobenartig die Qualität von routinemäßig angefertigten Kopf- und Halssonographiebefunden nach Kriterien der KVB an einer Auswahl deutscher HNO-Universitätskliniken.

## Studiendesign und Untersuchungsmethoden

Insgesamt wurden 70 zufällig ausgewählte, anonymisierte schriftliche Befunde einschließlich des zugehörigen Bildmaterials von insgesamt 7 HNO-Universitätskliniken (Erlangen, Freiburg, Homburg, Jena, Mainz, Mannheim, Regensburg) nach KV-Kriterien durch einen erfahrenen Prüfer der KV Bayern (KFH) retrospektiv ausgewertet. Die Anfrage zur Studienteilnahme der Zentren erfolgte nicht systematisch, sondern selektiv vorwiegend auf der Basis einer Sitzung der Kopf- und Halssektion der DEGUM. Jede der teilnehmenden Kliniken stellte 10 Befunde aus der allgemeinen Klinikroutine zur Auswertung bereit. Allen teilnehmenden Kliniken stehen hochmoderne Ultraschallgeräte zur Verfügung. Die HNO-Universitätskliniken Homburg und Freiburg sind DEGUM-zertifizierte Zentren und verfügen über DEGUM-Kursleiter der Stufe III. Erlangen, Mainz und Regensburg verfügen über Mitarbeiter mit der DEGUM-Stufe III und Jena über Stufe-IIb-Kursleiter. In Erlangen, Freiburg und Mainz wird ein DEGUM-zertifizierter Ausbildungskurs der Kopf- und Halssonographie angeboten. Darüber hinaus beschäftigen alle diese Kliniken weitere Mitarbeiter mit DEGUM-Stufenzertifizierung.

Folgende 16 Parameter wurden für die Qualitätsbewertung der Dokumentation zugrunde gelegt: Fragestellung nicht erkennbar; Schnittebenen nicht nachvollziehbar; anatomische Bezeichnungen falsch/fehlen; pathologischer Befund nicht markiert; zweite Ebene fehlt; Messmarker fehlen/unvollständig; Messwerte unvollständig/fehlen; Befund kann nicht belegt werden; Bilddokumentation überstrahlt; Bilddokumentation unterstrahlt; Seitenbezeichnung fehlt; Kontrast nicht ausreichend; Eindringtiefe unzureichend; Befundbeschreibung lückenhaft; Diagnose fehlt/falsch; therapeutische/diagnostische Konsequenz fehlt.

In der Ultraschallvereinbarung heißt es in § 10 im Wortlaut, dass „bei pathologischem Befund eine Darstellung in 2 Schnittebenen oder – wenn dies nicht möglich ist – in einer Schnittebene (nur bei B‑Modus)“ mit „zur Befunderstellung notwendigen Messwerten und Messmarkern“ gefordert wird. Im Rahmen der vorliegenden Auswertung wurden für eine korrekte Befundung in jeder Bildebene 2 Messmarker bzw. 2 Messwerte vorausgesetzt. Sofern also beispielsweise ein Befund nicht in 2 Ebenen markiert ist und/oder eine der Ebenen nicht in 2 Orientierungen markiert wurde, sind die Messmarker fehlerhaft oder unvollständig und folglich auch die Messwerte unvollständig oder fehlend. In der Diskussion dieser Arbeit wird dieser Sachverhalt vertiefend erörtert.

Die Datenauswertung erfolgte deskriptiv. Auf eine exakte Darstellung der Einzelergebnisse nach Klinik wurde aus Diskretionsgründen verzichtet. Aufgrund des rein retrospektiven Charakters der Studie ohne Patientenbezug wurde kein Ethikvotum beantragt. Die Studie erfolgte unter Einhaltung der Vorgaben der aktuellen Fassung der Deklaration von Helsinki.

## Ergebnisse

Die Art der Bild- und Befunddokumentation sowie die Archivierungsmethode in den einzelnen Kliniken ist in Tab. 1 dargestellt.

**Table 1:** 

Standort	Befund	Bild	Struktur	Textbausteine	Befund und Bild Kombination
Erlangen	Digital	Digital	Ja	Ja	KIS/PACS integriert
Freiburg	Papier	Thermoprint	Nein	Nein	Tacker
Homburg	Digital	Digital	Ja	Ja	KIS/PACS integriert
Jena	Digital	Digital	Ja	Ja	KIS/PACS separiert
Mainz	Digital	Digital	Nein	Nein	KIS/PACS separiert
Mannheim	Papier	Thermoprint	Nein	Nein	Tacker
Regensburg	Papier	Thermoprint	Nein	Nein	Tacker

*KIS* Klinikinformationssystem, *PACS* Bildarchivierungs- und Kommunikationssystem

Von den 70 Befunden konnten 69 in die vorliegende Studie eingeschlossen und ausgewertet werden. Lediglich ein Befund musste aufgrund schlechter Druckqualität der Befundkopie von der Auswertung ausgeschlossen werden. Die Dokumentationsvollständigkeit der gesamten Stichprobe variierte von 50–100 % mit einem Mittelwert von 80,6 % (Standardabweichung 14,5 %). Insgesamt waren 9 Befunde vollständig und korrekt dokumentiert (13 %).

Die Dokumentationsvollständigkeit der einzelnen Kliniken lag aufsteigend sortiert bei 68,1 %, 73,2 %, 79,4 %, 79,4 %, 81,9 %, 90,7 % und 93 %.

Die Häufigkeiten der Mängel in Bezug auf die einzelnen ausgewerteten Befundparameter ist in Tab. 2 dargestellt.

**Table 2:** 

Fragestellung nicht erkennbar	15,9
Schnittebenen nicht nachvollziehbar	20,3
Anatomische Bezeichnungen falsch/fehlen	42
Pathologischer Befund nicht markiert	10,1
Zweite Ebene fehlt	20,3
Messmarker fehlen/unvollständig	40,6
Messwerte fehlen/unvollständig	52,2
Befund kann nicht belegt werden	20,3
Bilddokumentation überstrahlt	4,3
Bilddokumentation unterstrahlt	2,9
Seitenbezeichnung fehlt	11,6
Kontrast nicht ausreichend	10,1
Eindringtiefe unzureichend	11,6
Befundbeschreibung lückenhaft	8,7
Diagnose fehlt/falsch	10,1
Therapeutische/diagnostische Konsequenz fehlt	28,6

**Figure 1:**
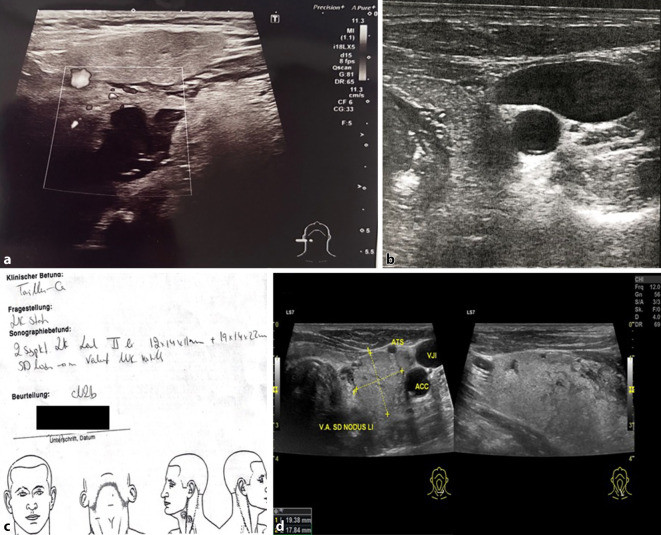

Es zeigt sich, dass mit einer Häufigkeit von über 25 % besonders die Befundbestandteile „therapeutische/diagnostische Konsequenz“, „Messmarker und Messwerte“ und „anatomische Bezeichnungen“ fehlerhaft bzw. unvollständig waren. In Abb. 1a–d sind exemplarisch derartige Befunde veranschaulicht.

Unter Berücksichtigung der Verwendung eines strukturierten Befundes und Textbausteinen ergeben sich für die Dokumentationsvollständigkeit unterschiedliche Mittelwerte. Mit 88,5 % vs. 75 % zeigten die Kliniken mit strukturierter Erhebung eine höhere Befundvollständigkeit. In 75 % der Fälle verfügten die Kliniken mit strukturiertem Dokumentationsprozess ebenfalls über digitale Lösungen zur Befundung und Bildarchivierung.

## Diskussion

Die Ultraschalldiagnostik im Kopf- und Halsbereich ist seit Jahrzehnten eine fest etablierte Untersuchungsmethode für eine Vielfalt an Kopf- und Halspathologien. Gerade zur Beurteilung von Hals- oder Speicheldrüsenraumforderungen, beim Primär- oder Restaging von Kopf- und Halsmalignomen oder in der Tumornachsorge müssen hohe Anforderungen an die Befunddokumentation und Archivierung gestellt werden [2, 11, 12, 16]. Die exakte topographische Zuordnung der Pathologie im Ultraschall ergibt sich für den nachbehandelnden Arzt erst komplementär aus der Bildbetrachtung mit dem zugehörigen schriftlichen Befund, denn im Gegensatz zur sonstigen klassischen Schnittbildgebung kann hier durch einfaches „Bildscrollen“ primär kein räumlicher Eindruck vermittelt werden. Die modernen Ultraschallgeräte bieten zwar weitgehend die Möglichkeit zur Videodokumentation, doch diese eigentlich optimale Dokumentationsvariante ist wegen häufig noch fehlender Schnittstellenanbindung an die PACS-Systeme und teilweise limitierten Speicherkapazitäten für Videos bisher kaum in der Praxis angekommen. Die fehlende Implementierung der Ultraschallbefunde in das KIS/PACS verhindert eine Demonstration von Ultraschallbefunden und -videos im Rahmen von interdisziplinären Boards (Röntgendemonstration, Tumorkonferenz). Dies verringert aus rein technischen Gründen die Wertigkeit bzw. die Bedeutung der sonographischen Diagnostik gerade im interdisziplinären Diskurs im Vergleich zu den klassischen Schnittbildverfahren. Die Möglichkeit zur Korrelation von Ultraschallbefunden mit CT-/MRT-Aufnahmen ist häufig nicht in gleicher Qualität möglich. Da die Sonographie eine höhere Auflösung und Genauigkeit in der Lymphknotenbeurteilung als konventionelle Schnittbildverfahren aufweist, könnte die angesprochene Implementierung von Ultraschallbefunden somit zur weiteren Optimierung der Tumorboardentscheidungen beitragen [12].

Eine wesentliche Limitation der Studie ist, dass diese nur stichprobenartig die Art und die Qualität der Befunddokumentation in einer selektionierten Gruppe von Universitätsklinika abbildet. Rückschlüsse auf die Dokumentationsqualität in anderen universitären Häusern oder nichtuniversitären HNO-Abteilungen lassen unsere Daten nicht zu. Auch ohne statistische Überprüfung bzw. ohne Kontrollgruppe zeigen die vorliegenden Daten, dass selbst in einem selektionierten Kollektiv aus vorwiegend HNO-Universitätskliniken mit objektiv hoher Ultraschallexpertise gut 80 % der Befunde nach den Kriterien der KV Bayern beanstandet wurden [13]. Lediglich 13 % der Befunde gingen vollständig mit der hier angewendeten Auslegung der KV-Vereinbarung konform.

Allerdings ist davon auszugehen, dass in Bezug auf „Messmarker/Messwerte“ die Ergebnisse besser ausgefallen wären, wenn die klinisch übliche Messmarkierung in 2 Ebenen mit 3 Orientierungen als unkritisch korrekt klassifiziert worden wäre. Letztlich wird hierbei allerdings angenommen, dass eine unregelmäßig gewachsene Raumforderung (z. B. polyzyklisches pleomorphes Adenom oder extranodal infiltrierende Lymphknotenmetastase) in 2 Ebenen mit 3 Orientierungen immer exakt erfasst wird. Die Vermessung einer Raumforderung in 2 um 90° gedrehten Ebenen ergibt nur dann für die kurze Achse jeweils den gleichen Wert, wenn der Schallkopf exakt auf einem Punkt gedreht wird und dort die dritte Achse vermessen wird. Häufig werden in der Realität bewusst oder unbewusst die jeweiligen Maximaldurchmesser vermessen, die nicht unbedingt im 90°-Winkel zueinander stehen. In diesen Fällen generiert die zweite Orientierung in der zweiten Ebene mutmaßlich eine zusätzliche Information, die nicht a priori vernachlässigt werden sollte.

Ein benigner Lymphknoten zeichnet sich im Ultraschall durch eine glatt begrenzte, ovaläre Form mit einem Lymphknotendurchmesser von < 10 mm (kurze Achse) aus [7]. Ein weiteres Kriterium zur Differenzierung zwischen „benigne“ und „maligne“ ist mit dem Index der langen zur kurzen Achse des Lymphknotens beschrieben („Solbiati-Index“). Nach Steinkamp et al. lag die Sensitivität bei 95 % bei einem „Cut-off-Wert“ des Index von 2 [20]. Folglich gilt in der Routine als Faustregel: Je runder der Lymphknoten ist, desto wahrscheinlicher liegt ein maligner Befund vor (Solbiati-Index < 2). Die Lymphknotenform sollte jedoch nicht allein als Differenzierungskriterium verwendet werden, da benigne submandibuläre, im Mundboden (Level IA) gelegene, und intraparotideale Lymphknoten häufig eine rundliche Form haben [22]. Für die Parameter „kurze Achse“ und „Solbiati-Index“ ist die Darstellung in einer zweiten Ebene sogar gänzlich entbehrlich. Die Darstellung einer Raumforderung in 2 Ebenen dient allerdings insgesamt der besseren Reproduzierbarkeit. Es besteht wenig Evidenz für die Vermessung einer Raumforderung in 3 Orientierungen. Eine Volumenmessung ähnlich der Volumetrie der Schilddrüse ist lediglich eine mathematische Annäherung unter der Annahme eines ellipsoiden Körpers, was letztlich nicht generell für zervikale Raumforderungen präjudiziert werden kann. Sofern sinnvollerweise Maximaldurchmesser gemessen werden, ist nicht von einer exakt um 90° verdrehten Messachse auszugehen und somit auch eine Vermessung der zweiten Ebene in 2 Orientierungen zu fordern. Noch genauer wäre formal eine Mehrfachvermessung einer Raumforderung mit Minimal- und Maximaldurchmessern in 2 Ebenen und 4 Orientierungen.

Zusammenfassend zeigen die Ergebnisse, dass in etwa 50 % der Befunde Messmarker und Messwerte nicht korrekt gesetzt wurden, wobei entweder eine zweite Ebene gänzlich fehlte (ca. 20 %) oder die zweite Ebene gar nicht oder nur in einer Orientierung vermessen wurde (Abb. 1d). Im Rahmen einer klinischen Studie sollten die im B‑Scan verfügbaren Messparameter zervikaler Raumforderungen (z. B. kurze Achse, Solbiati-Index, Maximaldurchmesser, Min.-Max.-Durchmesser, 90° gedrehte zweite Ebene) weiter evaluiert und letztlich validiert werden. Die gängige Praxis der Vermessung einer Raumforderung in 2 Ebenen mit 3 mehr oder weniger zufällig ausgewählten Orientierungen sollte jedenfalls kritisch hinterfragt werden. Für die klinische Routine mit zeiteffektiver Befunderstellung sollte die Vermessung von Raumforderungen sinnvoll und gut reproduzierbar, gleichzeitig aber auch möglichst wenig fehleranfällig sein.

Sämtliche Befundparameter können in einer strukturierten digitalen Befundmaske vollständig vorgegeben, mittels Textbausteinen bzw. Drop-down-Menüs befüllt und gerade häufig beanstandete Befundfelder „therapeutische/diagnostische Konsequenz“, „Messmarker und Messwerte“ als Pflichtfelder abgefragt werden. Anatomische Bezeichnungen können zwar an den Ultraschallgeräten vorbelegt werden, sind dann jedoch aktiv vom Untersucher einzupflegen.

Die strukturierte Dokumentation hat sich als vielversprechender Ansatz zur Standardisierung des Befundes und zur Verbesserung der Gesamtberichtqualität verschiedener Diagnosemodalitäten, einschließlich Kopf- und Halsultraschall, erwiesen [3, 6, 14, 19, 21]. Darüber hinaus bevorzugen überweisende und untersuchende Kollegen im Allgemeinen strukturierte Berichte gegenüber Freitextbefunden [1, 8, 15]. Da der Ultraschall ein Schlüsselelement bei der ambulanten Tumornachsorge und Operationsplanung ist, sind umfassende und verständliche Berichte unabdingbar. Der Befund jeglicher bildgebenden Untersuchung stellt die Essenz der Informationsweitergabe dar. Vollständige und zweifelsfrei interpretierbare Ausgangsbefunde sind die Grundlage für Kontrolluntersuchungen und für den nachfolgenden Untersucher zur Bewertung etwaiger Veränderungen unabdingbar. Eine strukturierte Befunderhebung und Dokumentation ist im Rahmen der Fachartausbildung bisher kein Ausbildungsstandard, wird allerdings im Curriculum der DEGUM-zertifizierten Ultraschallkurse adressiert und ist letztlich Qualitäts- und Zertifizierungsmerkmal der KV-Ultraschallvereinbarung. Letztere setzt die Empfehlungen und Vorgaben der DEGUM in der Ultraschallvereinbarung um.

Insbesondere unerfahrene Untersucher können von der Verwendung einer strukturierten Dokumentation profitieren, da während Erstellung des Befundberichtes auf relevante Inhalte sowie die Benennung anatomischer Strukturen hingewiesen und die empfohlene Terminologie angeboten wird [4, 5]. Leitenden Ärzten obliegt es, die Befunderstellung und die -qualität während der Facharztausbildung zu supervidieren.

Unsere Daten zeigen, dass die strukturierte Befunderhebung ggf. unter Verwendung von Textbausteinen mit einer Verbesserung der Dokumentationsvollständigkeit einhergeht (88,5 % vs. 75 %). Die Ergebnisse lassen den zusätzlichen Nutzen von standardisierter digitaler Befunderhebung und Archivierung erkennen. Die Einführung und Umsetzung der dargestellten Maßnahmen sollten daher dringend angestrebt werden. Erkennbar wird, dass Handlungsbedarf besteht, denn nur 60 % der an der Studie teilnehmenden Abteilungen dokumentieren digital, und nur 2 der mitwirkenden Kliniken verfügen über eine integrierte digitale Befund- und Bilddokumentation. An die erfolgreiche Einführung digitaler Lösungen besteht ein hoher Anspruch, denn sie müssen eine hohe und variable Schnittstellenkompatibilität aufweisen, den technischen Support garantieren, einfach, schnell und intuitiv in der Anwendung sein sowie die Anpassung durch den Benutzer ermöglichen.

## Fazit für die Praxis

Die Befundvollständigkeit und -qualität von routinemäßig angefertigten Kopf- und Halssonographiebefunden an einer Auswahl von HNO-Universitätskliniken ist insgesamt optimierbar. Die Implementierung strukturierter Befundmasken, die Umstellung der analogen Dokumentation auf digitale Lösungen sowie die Vernetzung in das KIS/PACS der Kliniken sollte weiter gefördert werden. Leitende Ärzte in Kliniken sind dazu angehalten, die Befundqualität unerfahrener Kollegen regelmäßig zu prüfen und auf die Einhaltung entsprechender Standards zu achten.

## References

[1] Armbruster M, Gassenmaier S, Haack M (2018). Structured reporting in petrous bone MRI examinations: impact on report completeness and quality. Int J Comput Assist Radiol Surg.

[2] Beltz A, Gösswein D, Zimmer S (2018). Staging of oropharyngeal carcinomas: new TNM classification as a challenge for head and neck cancer centers. HNO.

[3] Ernst BP, Hodeib M, Strieth S (2019). Structured reporting of head and neck ultrasound examinations. BMC Med Imaging.

[4] Ernst BP, Katzer F, Künzel J (2019). Impact of structured reporting on developing head and neck ultrasound skills. BMC Med Educ.

[5] Ernst BP, Strieth S, Katzer F (2020). The use of structured reporting of head and neck ultrasound ensures time-efficiency and report quality during residency. Eur Arch Otorhinolaryngol.

[6] European Society of Radiology (2018). ESR paper on structured reporting in radiology. Insights Imaging.

[7] Furukawa MK, Furukawa M (2010). Diagnosis of lymph node metastases of head and neck cancer and evaluation of effects of chemoradiotherapy using ultrasonography. Int J Clin Oncol.

[8] Gassenmaier S, Armbruster M, Haasters F (2017). Structured reporting of MRI of the shoulder—improvement of report quality?. Eur Radiol.

[9] Heyduck A, Jecker P, Bozzato A (2018). A german-wide inquiry about the significance of ultrasound in the head and neck area. Laryngorhinootologie.

[10] Kim SH, Sobez LM, Spiro JE (2020). Structured reporting has the potential to reduce reporting times of dual-energy x-ray absorptiometry exams. BMC Musculoskelet Disord.

[11] Künzel J, Bozzato A, Strieth S (2017). Follow-up ultrasound of head and neck cancer. HNO.

[12] Künzel J, Strieth S, Wirth G (2018). Ultrasound in the re-staging of cervical metastases after chemoradiotherapy for head and neck cancer. Ultraschall Med.

[13] KVB (2020). Vereinbarung von Qualitätssicherungsmaßnahmen nach § 135 Abs. 2 SGB V zur Ultraschalldiagnostik.

[14] Morgan TA, Helibrun ME, Kahn CE (2014). Reporting initiative of the Radiological Society of North America: progress and new directions. Radiology.

[15] Nörenberg D, Sommer WH, Thasler W (2017). Structured reporting of rectal magnetic resonance imaging in suspected primary rectal cancer: potential benefits for surgical planning and interdisciplinary communication. Invest Radiol.

[16] Psychogios G, Rueger H, Jering M (2019). Ultrasound can help to indirectly predict contact of parotid tumors to the facial nerve, correct intraglandular localization, and appropriate surgical technique. Head Neck.

[17] Sabel BO, Plum JL, Kneidinger N (2017). Structured reporting of CT examinations in acute pulmonary embolism. J Cardiovasc Comput Tomogr.

[18] Schoeppe F, Sommer WH, Nörenberg D (2018). Structured reporting adds clinical value in primary CT staging of diffuse large B-cell lymphoma. Eur Radiol.

[19] Schöppe F, Sommer WH, Schmidutz F (2018). Structured reporting of x-rays for atraumatic shoulder pain: advantages over free text?. BMC Med Imaging.

[20] Steinkamp HJ, Cornehl M, Hosten N (1995). Cervical lymphadenopathy: ratio of long- to short-axis diameter as a predictor of malignancy. Br J Radiol.

[21] Tuncyurek O, Garces-Descovich A, Jaramillo-Cardoso A (2019). Structured versus narrative reporting of pelvic MRI in perianal fistulizing disease: impact on clarity, completeness, and surgical planning. Abdom Radiol (NY).

[22] Ying M, Ahuja A, Brook F (1996). Sonographic appearance and distribution of normal cervical lymph nodes in a Chinese population. J Ultrasound Med.

